# Supplementary material for: Invasive goldfish (Carassius auratus) maintain aerobic scope across acute warm water temperatures
Source: Biol Open. 2025 Sep 3;14(9):bio062160. doi: 10.1242/bio.062160 (PMC12444859; doi:10.1242/bio.062160)
Supplement: Supplementary information [file biolopen-14-062160-s1.pdf]

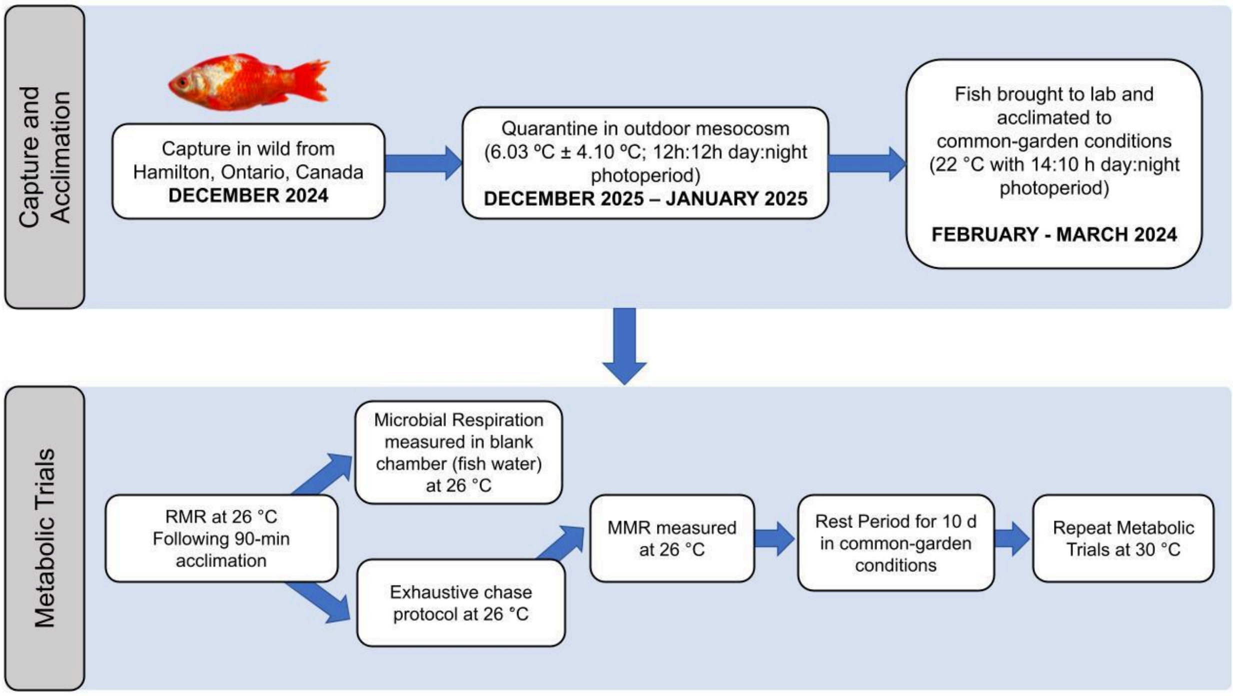

Fig. S1. Flowchart of experimental timelines. Fish were first captured in December 2024 from an urban stormwater pond in Hamilton, ON. Per facility regulations, they were quarantined for a period of 2 months in a semi-natural outdoor mesocosm in ambient conditions (6.03 °C average temperatures with a 12:12h day:night photoperiod). In February 2025 they were brought into the lab and warmed to lab temperatures of 22 °C at a rate of 0.02 °C/min, then acclimated to common-garden "Summer" lab conditions (constant 22 °C, 14:10h day:night photoperiod) for 3 weeks. In mid-March 2025, we began metabolic trials. We first tested all fish at 26 °C for routine metabolic rate (RMR) and maximum metabolic rate (MMR), recording background respiration in individual chambers between RMR and MMR tests. Each tested fish rested for 10 d under the 22 °C common-garden conditions before being retested for RMR and MMR at 30 °C.

Biology Open • Supplementary information

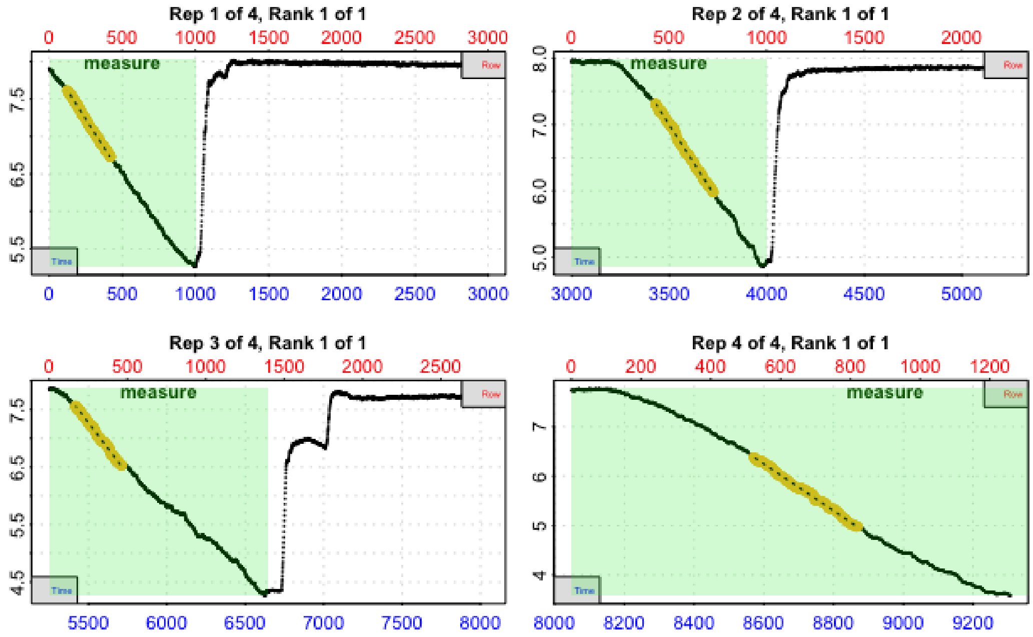

Fig. S2. Representative example of how an appropriate acclimation period was determined in pilot trials. The plots above represent four recording phases, separated by flush phases, of an individual Goldfish (*Carassius auratus*) in a respirometer. A continuous log was taken over 3 h of repeated flushing and recording for each pilot trial, for a total of 16 trials. The *auto_rate* function was used in the respR package to determine 5-min intervals in which the lowest oxygen consumption occurred with high stability (R^2>0.95) for each 3 h trial. After applying the rolling regression to all trials, we determined that the lowest, stable rate of oxygen consumption occurred, on average, 72 min after introducing fish to the respirometer. We therefore conservatively used an acclimation period of 90 min.

Biology Open • Supplementary information

Table S1. Data and/or descriptions of methodologies used to estimate routine and maximum metabolic rates and aerobic scope using respirometry, based on Killen et al. (2021), in an experiment using feral, invasive Goldfish (*Carassius auratus*) tested using static-type respirometry at acute peak summer temperatures of 26 and 30 °C.

Number	Criteria	Reporting data and/or descriptions
Equipment, materials and setup		
**1**	Body mass of animals at time of respirometry	Fish were weighed immediately after respirometry trials (each fish tested was individually weighed the day of testing). Mass data are reported in the supplemental data file; average fish mass was 5.76 g.
**2**	Volume of empty respirometer (in grams)	280 mL
**3**	How chamber mixing was achieved	Achieved through a stir bar placed inside each chamber. The chambers were placed on top of magnetic stir plates to initiate gentle stirring.
**4**	Ratio of net respirometer volume (plus any associated tubing in mixing circuit) to animal body mass	Average mass of 5.76 g; 49 times the volume of the fish; range: 31–112 times fish volume. No additional tubing was used (static-type design).
**5**	Material of tubing used in any mixing circuit	Plastic RO system tubing used to flush static respirometers with fresh water during acclimation.
**6**	Volume of tubing in any mixing circuit	N/A
**7**	Confirm volume of tubing in any mixing circuit is included in calculations of oxygen uptake rates	N/A

Biology Open • Supplementary information

**8**	Material of respirometer	Glass
**9**	Type of oxygen probe and data recording	Pyroscience FireStingO2 optical oxygen meter reading from OXSP5 contactless sensor spots using the SPFIB-BARE fiber optic cable. Data recorded on Windows 10 PC using Pyro Firesting Logger software (Pyroscience GmbH, Aachen, Germany).
**10**	Sampling frequency of water-dissolved oxygen	1 s
**11**	Placement of oxygen probe	Directly in the chamber
**12**	Flow rate during flushing and recirculation, or confirm that chamber returned to normoxia during flushing	Chambers had ~100% DO during flushing and at onset of valve sealing for recording.
**13**	Timing of flush/closed cycles	N/A; flushing was only done during the acclimation period.
**14**	Wait (delay) time excluded from closed measurement cycles	N/A
**15**	Frequency and method of probe calibration (for both 0 and 100% calibrations)	Frequency: daily for both 0% and 100% calibrations<br><br>Method: a sodium sulphite solution in a concentration of 30g/L was used for 0% calibration as per manufacturer instructions.<br><br>Air saturated water was used for 100% calibration
**16**	Whether software temperature compensation was used during recording of water oxygen concentration	Automatic temperature compensation from Firesting temperature probe and Firesting Logger Software.
Measurement conditions		
**17**	Temperature during respirometry	26°C and 30°C ± 0.5 (target),

		actual mean temperatures during trials were:<br><br>26.44, 26.29, 26.21<br>30.09, 30.47, 30.26
**18**	How temperature was controlled	Water bath using pre-set heater; experiments began once temperatures were stabilized.
**19**	Photoperiod during respirometry	All respirometry experiments were done in the morning (14h:10h light:dark cycle) during the 14h light period.
**20**	If (and how) the ambient water bath was cleaned and aerated during measurement of oxygen uptake (e.g. filtration, periodic or continuous water changes)	Flushing occurred once during the acclimation period; prior to flushing, all equipment was sterilized with 70% ethanol.
**21**	Total volume of ambient water bath and any associated reservoirs	Water bath: 15 L<br>Reservoir: 19 L
**22**	Minimum water oxygen level reached during closed phases	~70% DO
**23**	Whether chambers were visually shielded from external disturbance	Chambers were shielded from each other and experimenters using opaque dividers. Opaque black rubber lids were placed on top of chambers.
**24**	How many animals were measured during a given respirometry trial (i.e. how many animals were in the same water bath)	One animal per respirometer, four respirometers measured in parallel.
**25**	If multiple animals were measured simultaneously, state whether they were able to see each other during measurements	Opaque dividers prevented the fish from seeing each other and the experimenters.
**26**	Duration of animal fasting before placement in respirometer	48 hours
**27**	Duration of all trials combined (number of days	16 days

	to measure all animals in the study)	
**28**	Acclimation time to the laboratory (or time since capture for field studies) before respirometry measurements	3 week lab acclimation
Background respiration		
**29**	Whether background microbial respiration was measured and accounted for, and if so, method used (e.g. parallel measures with empty respirometer, measurements before and after for all chambers while empty, both)	Measurements were taken for each individual chamber for each trial. Measurements lasted as long as the individual fish was being chased (~10-15 mins) following RMR but prior to MMR trials using fish water (*i.e.* the water inside the chamber that the fish was in). No parallel blank chamber was used to reduce inaccurate measurements.
**30**	If background respiration was measured at beginning and/or end, state how many slopes and for what duration	The entire slope was used for each background respiration recording (varied between 10-15 mins depending on how long the fish were chased), per chamber, per trial.
**31**	How changes in background respiration were modelled over time (e.g. linear, exponential, parallel measures)	The slope of a best-fit linear regression line over the entire background recording period was subtracted from fish respiration values (RMR and MMR).
**32**	Level of background respiration (e.g. as a percentage of SMR)	~1-2%
**33**	Method and frequency of system cleaning (e.g. system bleached between each trial, UV lamp)	70% ethanol sterilization of all equipment before each trial (daily)
Routine Metabolic Rate		
**34**	Acclimation time after transfer to chamber, or	90 minutes

	alternatively, time to reach beginning of metabolic rate measurements after introduction to chamber	
**35**	Time period, within a trial, over which oxygen uptake was measured (e.g. number of hours)	Approximately 15 minutes on average
**36**	What value was taken as SMR/RMR (e.g. quantile, mean of lowest 10%, mean of all values)	Quantile (q0.25) – average of 25% lowest MO2 measurements
**37**	Total number of slopes measured and used to derive metabolic rate (e.g. how much data were used to calculate quantiles)	The average number of intervals used was 19 (range: 16-27).
**38**	Whether any time periods were removed from calculations of SMR/RMR [e.g. data during acclimation, periods of high activity (e.g. daytime)]	Data were not taken from the ~90 minute acclimation period. MO2 values calculated below 80% DO (normoxia) were removed. Otherwise, all recorded values were used.
**39**	r2 threshold for slopes used for SMR/RMR (or mean r2)	N/A
**40**	Proportion of data removed due to being outliers below r2 threshold	N/A
Maximum Metabolic Rate		
**41**	When MMR was measured in relation to SMR	After RMR measurements
**42**	Method used (e.g. critical swimming speed respirometry, swim to exhaustion in swim tunnel, or chase to exhaustion in tank or respirometer)	Chase to exhaustion in a separate rectangular arena (53.34 cm x 38.10 cm x 17.78 cm) set to test temperature using a heater.
**43**	What value was taken as MMR (e.g. the highest oxygen uptake rate value after transfer, average of highest values)	The entire post-chase oxygen consumption trace in normoxia.
**44**	Length of activity challenge used for estimating MMR (e.g. duration and water velocity increment of swim test, duration of chase in minutes or until exhaustion, etc.)	Chasing until exhaustion. On average, this was 10 minutes for each fish.

Biology Open • Supplementary information

**45**	If MMR was measured post- exhaustion, state whether further air-exposure was added after exercise	No.
**46**	If MMR was measured post- exhaustion, provide time until transfer to chamber after exhaustion and time to start of oxygen uptake recording	A maximum of 15 seconds.
**47**	Duration of slopes used to calculate MMR (e.g. 1 min, 5 min, etc.)	Entire duration
**48**	Slope estimation method for MMR (e.g. rolling regression, sequential discrete time frames)	One slope for all values in normoxia (80-100% DO) using calc_MO2 function of the *respirometry* package.
**49**	How absolute aerobic scope and/or factorial aerobic scope is calculated (i.e. using raw SMR and MMR, allometrically mass-adjusted SMR and MMR, or allometrically mass-adjusting aerobic scope itself)	Using whole-organism MMR-RMR values. Whole-organism values were used for modelling, with mass as a covariate to account for mass scaling. Mass-standardization (to an average 5.76 g fish) was done to create Figure 1 using model residuals.
	Data handling and statistics	
**50**	Sample size	24
**51**	How oxygen uptake rates were calculated (software or script, equation, units, etc.)	*calc_MO2* function, but detailed explanation is provided in methods.
**52**	Volume (or mass) of the animal was subtracted from respirometer volume when calculating oxygen uptake rates	Confirmed.
**53**	Whether variation in body mass was accounted for in analyses and describe any allometric body-mass- correction or adjustment	Confirmed (in model via mass covariate; in data figure via mass-standardization).

Biology Open • Supplementary information

Table S2. Data and metadata from metabolic experiments (routine metabolic rate [RMR], maximum metabolic rate [MMR], and aerobic scope [AS]) using feral, invasive Goldfish (*Carassius auratus*) tested at acute peak summer temperatures of 26 and 30 °C. Column headers: 'no' – unique identification for each individual trial; 'test.date' – date of trial; 'temperature.actual' – true mean temperature during trial (°C); 'treatment' – acute test temperature target (26 or 30 °C); 'tank' – tank of origin for test fish (of four replicate tanks); 'chamber' – unique identification for each of the 4 respirometers; 'fish.mass' – mass of each fish in g, measured after RMR trials; 'RMR', 'MMR', and 'AS' – whole-organism RMR. MMR, and AS, respectively; 'RMR.standardized', 'MMR.standardized', and 'AS.standardized' – mass-standardized RMR, MMR, and AS, respectively, adjusted to a 5.76 g fish using model residuals; 'notes' – explanation column for the two trials (no. 4 and 13) for which a faulty seal during MMR trials prevented us from estimating MMR and AS.

no	test.date	temperature.actual	treatment	tank	chamber	fish.mass	RMR	RMR.standardized	MMR	MMR.standardized	AS	AS.standardized	notes
**1**	March 15 2025	26.44	26	1	1	8.94	1.002246	1.379708	2.593531	2.011449	1.591285	1.192148	
**2**	March 15 2025	26.44	26	1	2	3.81	0.717033	1.427349	1.347828	1.776286	0.630795	0.902428	
**3**	March 15 2025	26.44	26	1	3	4.2	1.245145	1.922994	2.028667	2.365045	0.783522	0.998537	

Biology Open • Supplementary information

**4**	March 15 2025	26.44	26	1	4	6.22	0.879097	1.412298	NA	NA	NA	NA	Error during MMR (likely bad seal); no detectable decrease in MO2
**5**	March 16 2025	26.29339	26	3	1	6.87	0.708344	1.201128	2.113132	1.897231	1.404788	1.260514	
**6**	March 16 2025	26.29339	26	3	2	7.6	0.88136	1.331376	2.129354	1.779542	1.247994	1.01195	
**7**	March 16 2025	26.29339	26	3	3	5.42	1.05936	1.645917	2.65004	2.719657	1.59068	1.636188	
**8**	March 16 2025	26.29339	26	3	4	8.22	1.045429	1.461004	2.668949	2.209664	1.62352	1.311213	
**9**	March 17 2025	26.21	26	4	1	7.65	0.917084	1.364262	2.799456	2.440678	1.882372	1.640123	
**10**	March 17 2025	26.21	26	4	2	7.87	1.205319	1.640139	1.985354	1.587417	0.780035	0.5106	
**11**	March 17 2025	26.21	26	4	3	9.06	1.42871	1.8	2.213206	1.611075	0.784496	0.371056	
**12**	March 17 2025	26.21	26	4	4	6.07	1.417925	1.960805	2.34856	2.2866	0.930635	0.88971	

**13**	March 25 2025	30.09	30	1	1	3.81	1.094483	1.896405	NA	NA	NA	NA	Error during MMR (likely bad seal); no detectable decrease in MO2
**14**	March 25 2025	30.09	30	1	2	5	1.216166	1.912127	1.708538	1.882586	0.492372	0.59468	
**15**	March 25 2025	30.09	30	1	3	4.2	0.905391	1.670657	1.729882	2.099857	0.824491	1.038691	
**16**	March 25 2025	30.09	30	1	4	5	0.592956	1.288917	1.255972	1.43002	0.663016	0.765323	
**17**	March 26 2025	30.47	30	3	1	3.5	1.279557	2.112239	1.655489	2.210443	0.375932	0.692375	
**18**	March 26 2025	30.47	30	3	2	4	1.034954	1.818753	1.440316	1.861702	0.405362	0.648319	
**19**	March 26 2025	30.47	30	3	3	4.5	1.319588	2.058015	2.728615	3.023393	1.409027	1.580706	
**20**	March 26 2025	30.47	30	3	4	2.5	0.602619	1.547346	1.119995	1.969483	0.517376	0.989179	
**21**	March 27 2025	30.26	30	4	1	7.5	2.360481	2.87498	3.484348	3.119405	1.123867	0.901111	

**22**	March 27 2025	30.26	30	4	3	6.5	0.723 38	1.30535 7	2.958 896	2.798631	2.2355 17	2.139022
**23**	March 27 2025	30.26	30	4	4	4	0.566 372	1.35017 1	2.661 636	3.083022	2.0952 64	2.33822
**24**	March 27 2025	Fish mortality prior to experimental onset										

Biology Open • Supplementary information
